# Practical Asymmetric Synthesis of Sitagliptin Phosphate Monohydrate

**DOI:** 10.3390/molecules23061440

**Published:** 2018-06-13

**Authors:** Haoling Gao, Jiangang Yu, Chengsheng Ge, Qun Jiang

**Affiliations:** 1 College of Chemistry and Materials Engineering, Quzhou University, Quzhou 324000, China; xjguo631@sina.com (H.G.); jgyu@qzu.zj.cn (J.Y.); 2 College of Chemical Engineering, Zhejiang University of Technology, Hangzhou 310014, China; jiangqun1991@163.com

**Keywords:** sitagliptin phosphate monohydrate, aza-Michael/hemiacetal reaction, asymmetric synthesis

## Abstract

Optically pure sitagliptin phosphate monohydrate is efficiently and practically synthesized through a chiral hemiacetal as the key intermediate in 54% overall yield starting from (*E*)-4-(2,4,5-trifluorophenyl)but-2-enal and *N*-boc-protected hydroxylamine. The chiral hemiacetal fragment is constructed by a tandem aza-Michael/hemiacetal reaction catalyzed by an organocatalyst and the influence of acidity of Brønsted acid on tandem aza-Michael/hemiacetal reaction is researched in detail.

## 1. Introduction

Since its launch in 2006 by Merck, sitagliptin phosphate monohydrate, the first DPP-4 inhibitor (dipeptidyl peptidase 4), has been one of the best-selling orally active and safe agents for the treatment of T2DM (type 2 diabetes mellitus) [1,2,3]. Considerable efforts have been made on searching for a short, environmentally friendly, and economic asymmetric synthetic route toward sitagliptin phosphate monohydrate including Merck’s three-generation process. In this process, the key structure of sitagliptin was installed via rare-metal catalyzed asymmetric hydrogenation, biocatalytic asymmetric reduction or transaminase-catalyzed asymmetric synthesis [4,5]. Generally, most of synthetic routes including the latest reported methods focus on constructing key chiral fragment by employing diverse metal catalysts [6,7,8], chiral auxiliary [9,10,11,12] or chiral starting materials [13,14].

In the last decades, a variety of chiral amino acids can be made from hemiacetals through catalytic tandem aza-Michael/hemiacetal reaction, using organocatalyst in high yield and enantioselectivity [15,16,17,18,19]. As part of our efforts to develop efficient and practical access to stigliptin phosphate monohydrate, we herein report an efficient synthetic route to make sitagliptin phosphate monohydrate by means of chiral hemiacetal as the key intermediate.

## 2. Results and Discussion

**Scheme 1:**
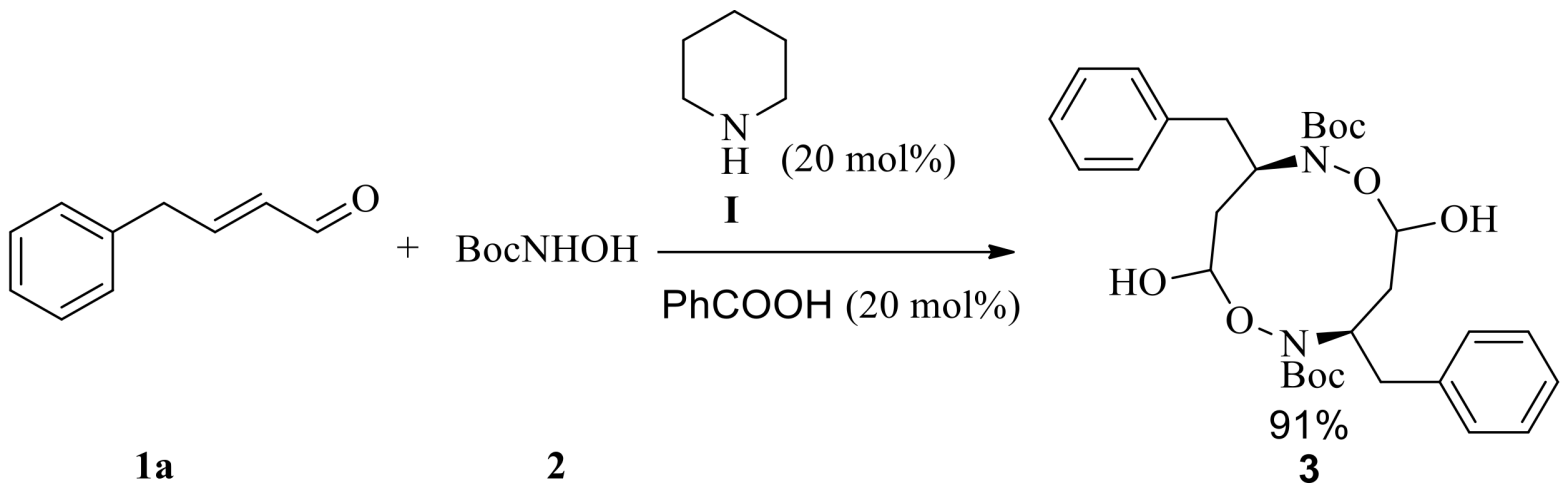
Tandem aza-Michael/hemiacetal reaction between (*E*)-4-phenyl)but-2-enal **1a** and *N*-Boc-protected hydroxylamine (**2**) catalyzed by piperidine (**I**) and benzoic acid to give 3,8-dibenzyl-5,10-dihydroxy-1,6,2,7-dioxadiazecane-2,7-dicarboxylate (**3**).

**Scheme 2:**
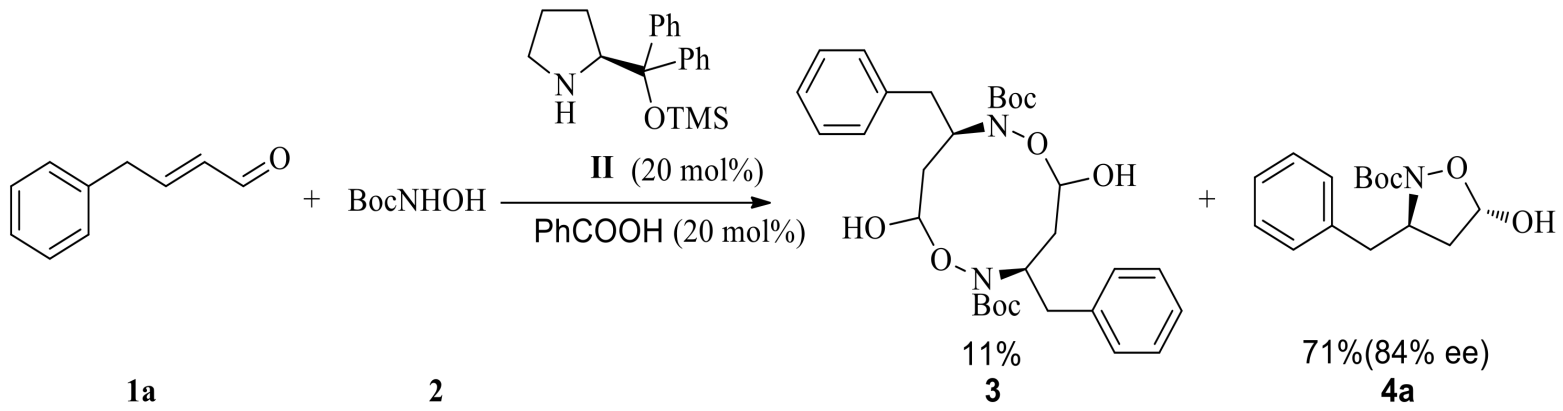
Tandem aza-Michael/hemiacetal reaction between (*E*)-4-phenyl)but-2-enal **1a** and *N*-Boc-protected hydroxylamine (**2**) catalyzed by catalyst **II** and benzoic acid to give 3,8-dibenzyl-5,10-dihydroxy-1,6,2,7-dioxadiazecane-2,7-dicarboxylate (**3**) and (3*R*,5*S*)-*tert*-butyl 3-benzyl-5-hydroxyisoxazolidine-2-carboxylate (**4a**).

Initially, the model aza-Michael addition reaction that we studied, between (*E*)-4-phenylbut-2-enal **1a** and *N*-Boc-protected hydroxylamine or *N*-Cbz-protected hydroxylamine catalyzed by organocatalyst I or II in chloroform at 0 °C did not take place and obtained the starting materials were only. However, when the mixture of 20 mol % benzoic acid (pKa = 4.19) [20] and 20 mol % piperidine were used as a catalyst, the tandem aza-Michael/hemiacetal reaction took place to give an unexpected macro-cyclic compound **3** in 91% isolated yield (Scheme 1). In contrast, macro-cyclic compound **3** and *N*-Boc hemiacetal **4a** was isolated in 11% and 71% yields, respectively, by using 20 mol % chiral secondary amine II together with 20 mol % benzoic acid as catalyst, and the enantiomeric excess (ee) of *N*-Boc hemiacetal **4a** up to 89% (Scheme 2). Obviously, the tandem aza-Michael/hemiacetal reaction adduct is different from the outcome of tandem aza-Michael/hemiacetal reaction between cinnamaldehyde and *N*-Boc-protected hydroxylamine in the literature with regard to distinguishable products [17]. We suppose that formyl group of aza-Michael addition adduct was activated by Brønsted acid (benzoic acid) to facilitate intramolecular hemiacetal formation to complete the tandem aza-Michael/hemiacetal reaction. We assume the acidity of Brønsted acid had great effect on the outcome of tandem aza-Michael/hemiacetal reaction.

**Scheme 3:**
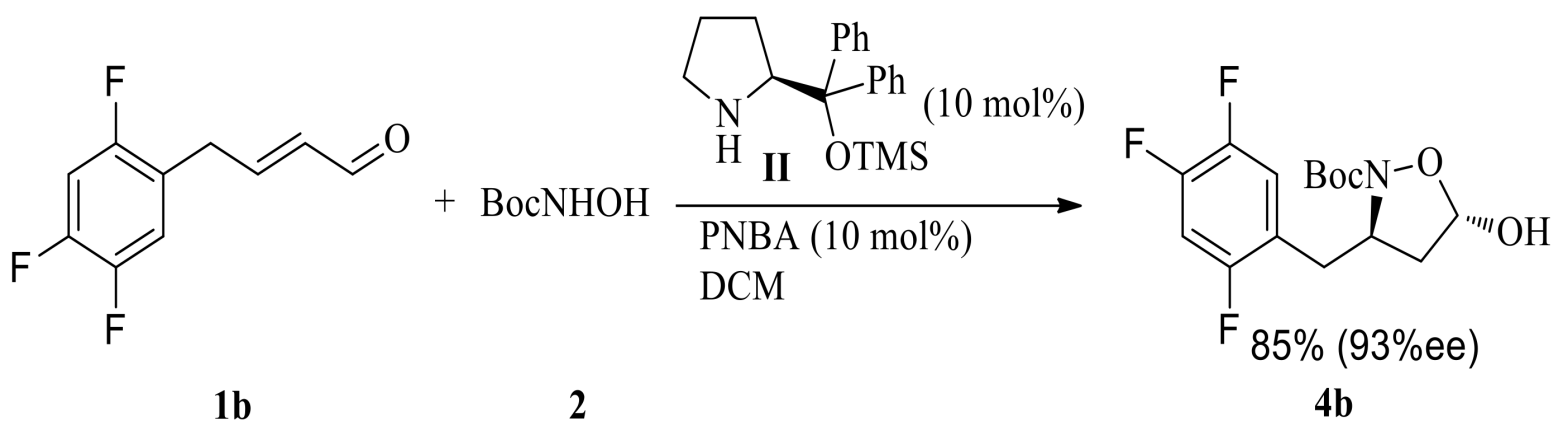
Tandem aza-Michael/hemiacetal reaction between (*E*)-4-(2,4,5-trifluorophenyl)but-2-enal **1b** and *N*-Boc-protected hydroxylamine (**2**) to give (3*R*,5*S*)-*tert*-butyl 5-hydroxy-3-(2,4,5-trifluorobenzyl)isoxazolidine-2-carboxylate (**4b**).

The evaluation on the influence of different Brønsted acid was shown in Table 1. When the stronger Brønsted acids, such as phosphoric acid (pKa = 2.12), trifluoroacetic acid (pKa = 0.23) and *p*-toluenesulfonic acid (pKa = −2.8), were used as additives, macro-cyclic compound **3** could not be found, however, *N*-Boc hemiacetal **4a** and double Boc derivative **5** were isolated. Meanwhile, the enantioselectivities were low at the presence of stronger Brønsted acids. Fortunately, hemiacetal **4a** was isolated as single product in 84% yield with 92% ee using 20 mol % *p*-nitrobenzoic acid (PNBA, pKa = 3.47) as an additive, together with 20 mol % (*S*)-diphenylprolinol-TMS II. It seems that Brønsted acid can effectively activate the formyl group of the aza-Michael addition adduct to facilitate the sequence, and acidity of Brønsted acid can mediate the outcome of tandem aza-Michael/hemiacetal reaction. In other solvents, such as diethyl ether, methanol, toluene, and dichloromethane, tandem aza-Michael/hemiacetal reaction catalyzed by (*S*)-diphenylprolinol-TMS II together with *p*-nitrobenzoic acid worked smoothly (Table 1, entries 5–8). Dichloromethane was the best solvent, as 90% yield and 92% ee can be obtained even when the loading amount of (*S*)-diphenylprolinol-TMS II and additive was reduced to 10 mol % (entry 9). Catalyst III and IV were also tested (entries 10–11), and it was found that catalyst III, containing a 3,5-ditrifluoromethylphenyl group, gave 45% ee, while catalyst IV afforded product in low yield with poor enantioselectivity.

(*E*)-4-(2,4,5-Trifluorophenyl)but-2-enal **1b** was obtained via either the cross-metathesis reaction between 1-allyl-2,4,5-trifluorobenzene and crotonaldehyde or the coupling reaction between 1-bromo-2,4,5-trifluorobenzene and (*E*)-4-bromo-1,1-dimethoxybut-2-ene [21]. Tandem aza-Michael/hemiacetal reaction between (*E*)-4-(2,4,5-trifluorophenyl)but-2-enal **1b** and *N*-Boc-hydroxylamine catalyzed by 10 mol % (*S*)-diphenylprolinol-TMS II and 10 mol % p-nitrobenzoic acid gave (3*R*,5*S*)-*N*-Boc-5-hydroxy-3-(2,4,5-trifluorobenzyl)isoxazolidine **4b** in 85% yield and 93% ee (Scheme 3). Decreasing the reaction temperature to −20 °C improved the enantioselectivty. The ee was up to 96% with an extended reaction time (15 h), and yield was up to 85%.

**Scheme 4:**
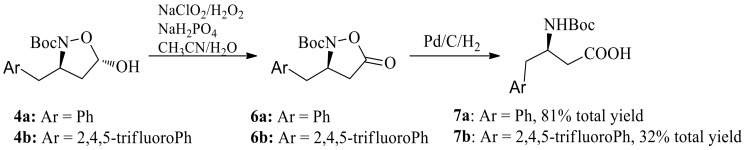
Oxidation/reductive cleavage of N-O bond consequence.

We next converted this key chiral unit to chiral *β*-amino acid. Oxidation/reductive cleavage of N-O bond sequence are a common methodology for this conversion. Chiral (*R*)-*N*-Boc-*β*-benzyl-*β*-amino acid **7a** was obtained via NaClO_2_/H_2_O_2_/NaH_2_PO_4_ oxidation/hydrogenation, starting from hemiacetal **4a** in 82% yield, without the loss of enantiomeric purity (Scheme 4). However, NaClO_2_/H_2_O_2_/NaH_2_PO_4_ oxidation/hydrogenation sequence starting from hemiacetal **4b** gave (*R*)-*N*-Boc-*β*-(2,4,5-trifluorobenzyl)-*β*-amino acid **7b** in poor yield (32% in total).

**Scheme 5:**
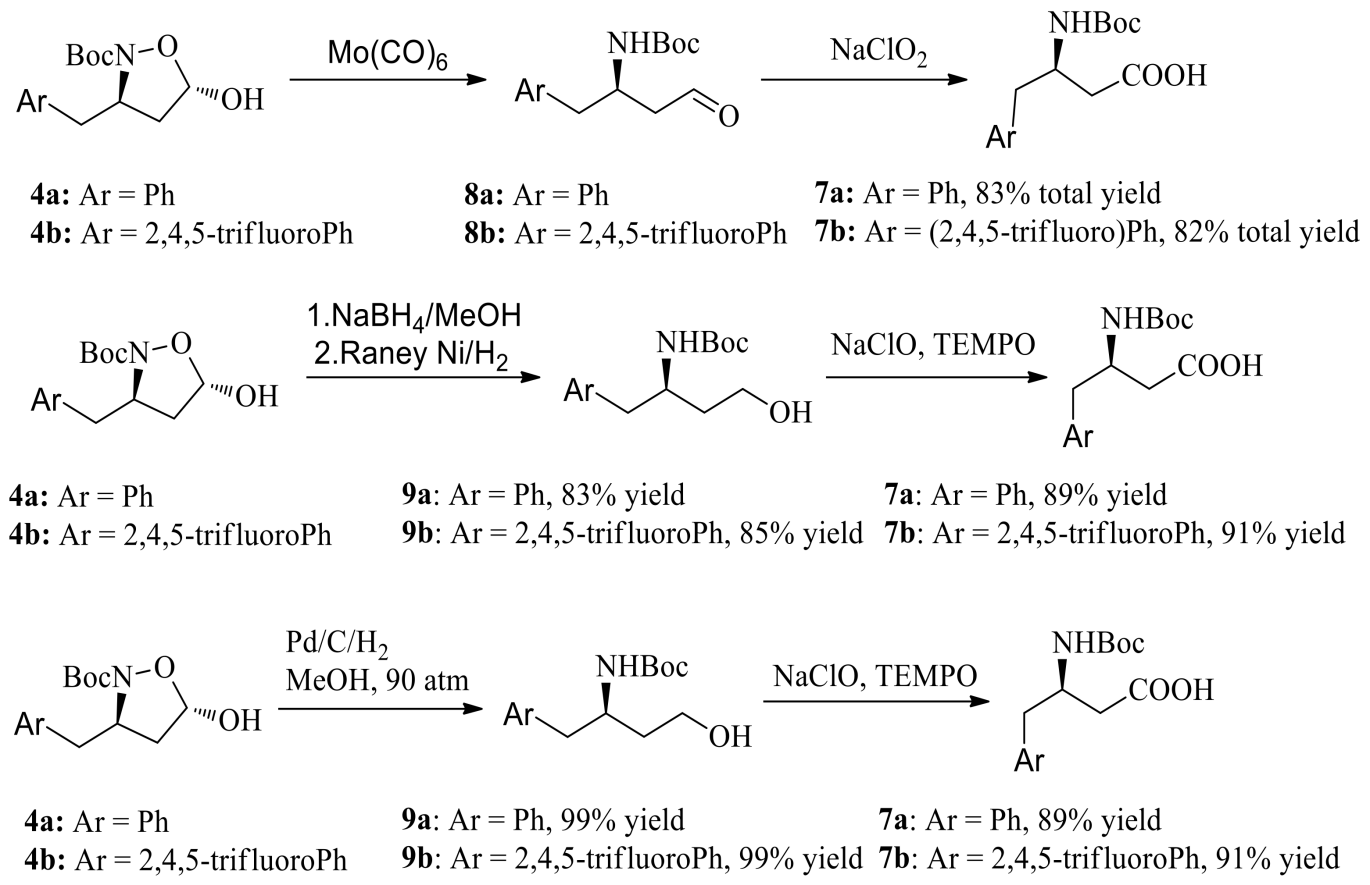
Reductive cleavage of N-O bond/oxidation sequence.

**Scheme 6:**
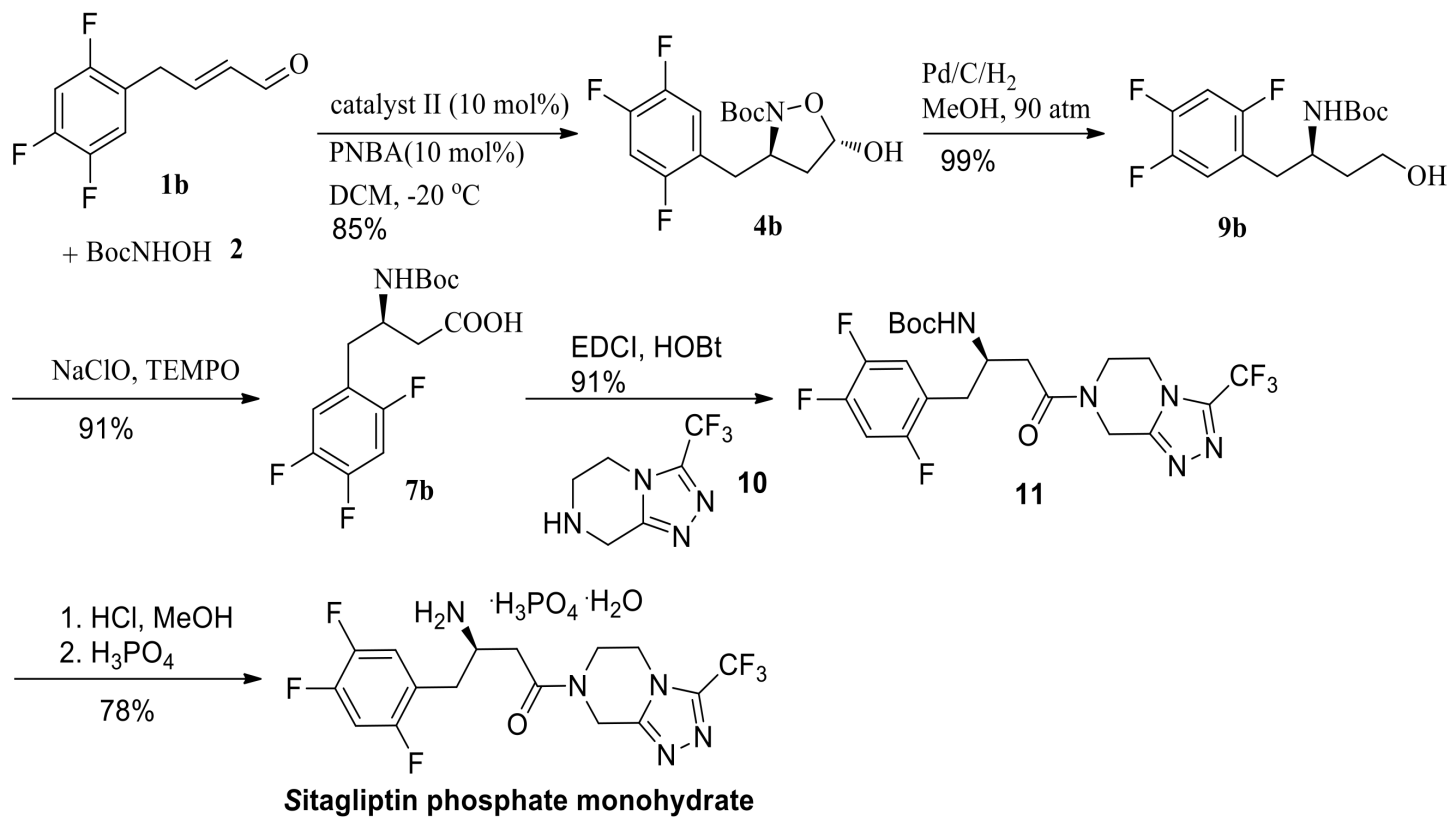
Synthesis of sitagliptin phosphate monohydrate.

We then turned our attention to obtain chiral *β*-amino acid via reductive cleavage of the N-O bond/oxidation sequence (Scheme 5). N-O bond reductive cleavage using Mo(CO)_6_ as the reducing agent, followed by oxidation, gave the *β*-amino acid **7a** and **7b** in 83% and 82% yields, respectively. Moreover, reduction with NaBH_4_, followed by N-O bond cleavage via hydrogenation with Raney Ni gave the *β*-amino alcohol **7a** and **7b** in 83% and 82% yields respectively. At a pressure of 90 atm of hydrogen and Pd/C, the *β*-amino alcohol **9a** and **9b** were obtained in 99% yield. Further oxidation of *β*-amino alcohol **9a** and **9b** with NaClO/TEMPO gave the *β*-amino acid **7a** and **7b** in 92% and 91% yield respectively. Compared with N-O bond reductive cleavage using expensive Mo(CO)_6_ or NaBH_4_/hydrogenation sequence, N-O bond reductive cleavage using hydrogenation have industrial potential.

(*R*)-*N*-Boc-*β*-(2,4,5-trifluorobenzyl)-*β*-amino acid **7b** was synthesized starting from (*E*)-4-(2,4,5-trifluorophenyl)but-2-enal in 76% total yield in a three-step procedure (Scheme 6). (*R*)-*tert*-butyl 4-oxo-4-(3-(trifluoromethyl)-5,6-dihydro-[1,2,4]triazolo[4,3-a]pyrazin-7(8H)-yl)-1-(2,4,5-tri-fluorophenyl) butan-2-ylcarbamate **11** was obtained in 91% yield via the coupling reaction between triazole (**10**) and (*R*)-*N*-Boc-*β*-(2,4,5-trifluorobenzyl)-*β*-amino acid **7b,** using HOBt-EDCI as the coupling agent. Finally, >99.9% ee of sitagliptin phosphate monohydrate was provided in 78% yield following the known literature procedure (as shown in Scheme 6) [6].

## 3. Materials and Methods

### 3.1. General

Unless otherwise noted, all commercial reagents were used as purchased from Aladdin and Tansoole (Chinese chemical reagents supplier). All the reactions were monitored by thin-layer chromatography (TLC) that was performed on silica gel plates GF254. Visualization was achieved under a UV lamp, and by developing the plates with Potassium Permanganate in water. Flash chromatography was performed using silica gel (200–300 mesh) with solvents indicated in the text. NMR spectra were registered in a Bruker Advance 400 Ultrashield spectrometer at room temperature, operating at 400 MHz (^1^H) and 100 MHz (^13^C). The experiments of high performance liquid chromatography (HPLC) was performed on a Shimadzu chromatograph (Essentia LC-16, Shimadzu Corp., Kyoto, Japan) by using Chiralcel columns as illustrated in supporting information.

### 3.2. Synthesis of (E)-4-Phenylbut-2-enal *(**1a**)*

A solution of allylbenzene (591 mg, 5 mmol) and crotonaldehyde (700 mg, 10 mmol) together with 0.1 mol % Grubbs-II catalyst (4.2 mg, 0.005 mmol) in dichloromethane (20 mL) was refluxed for 12 h, and then cooled to 0 °C. The mixture solution was concentrated under reduced pressure and purified over silica gel column chromatography (PE/EtOAc, 20:1–10:1) to give **1a** as a liquid (650 mg, 89%). ^1^H-NMR (400 MHz, CDCl_3_) δ 9.52 (d, *J* = 8.2 Hz, 1H), 7.34–7.31 (m, 2H), 7.27–7.23 (m, 1H), 7.17 (d, *J* = 8.5 Hz, 2H), 6.99–6.91 (m, 1H), 6.10 (dd, *J* = 8.1 Hz and 8 Hz, 1H), 3.64 (d, *J* = 4.2 Hz, 2H) (Supplementary Material Figure S1).

### 3.3. Synthesis of (E)-4-(2,4,5-Trifluorophenyl)but-2-enal *(**1b**)*

A solution of 1-allyl-2,4,5-trifluorobenzene (860 mg, 5 mmol) and crotonaldehyde (700 mg, 10 mmol), together with 0.1 mol % Grubbs-II catalyst (4.2 mg, 0.005 mmol) in dichloromethane (20 mL), was refluxed for 12 h, and then it was cooled to 0 °C. The mixture solution was concentrated under reduced pressure and purified over silica gel column chromatography (PE/EtOAc, 20:1–10:1) to give **1b** as a liquid (830 mg, 83%). ^1^H-NMR (400 MHz, CDCl_3_) δ 9.52 (d, *J* = 8.6 Hz, 1H), 7.02–6.83 (m, 3H), 6.08–6.02 (m, 1H), 3.60 (d, *J* = 4.4 Hz, 2H) (Supplementary Material Figure S2).

### 3.4. Synthesis of Di-tert-butyl 3,8-Dibenzyl-5,10-dihydroxy-1,6,2,7-dioxadiazecane-2,7-dicarboxylate *(**3**)*

(*E*)-4-phenylbut-2-enal **1a** (146 mg, 1 mmol) was dissolved in dichloromethane (5 mL), and then *N*-Boc-hydroxylamine **2** (199 mg, 1.5 mmol), piperidine (17 mg, 0.2 mmol), and benzoic acid (24 mg, 0.2 mmol) were added to the resulting solution at 0 °C. The reaction mixture was stirred for 8 h at 0 °C. The mixture solution was diluted with dichloromethane (20 mL) and washed with saturated sodium bicarbonate aqueous solution (5 mL) and brine (5 mL). The resulting organic phase was dried over Na_2_SO_4_, filtered and concentrated under reduced pressure. The crude product was purified over silica gel column chromatography (PE/EtOAc, 10:1–4:1) to give **3** as a white solid (254 mg, 91%). mp: 123–124 °C; ^1^H-NMR (400 MHz, CDCl_3_) δ 7.32–7.22 (m, 10H), 5.73–5.69 (m, 2H), 4.66–4.59 (m, 1H), 4.31–4.24 (m, 1H), 3.43 (s, 1H), 3.21–3.17 (m, 2H), 3.11 (dd, *J* = 4.2 Hz, 12 Hz, 1H), 2.94 (dd, *J* = 4.2 Hz, 12 Hz, 1H), 2.74 (dd, *J* = 4.1 Hz, 12 Hz, 1H), 2.58–2.51 (m, 1H), 2.24 (dd, *J* = 4.1 Hz, 12 Hz, 1H), 2.16–2.09 (m, 1H), 2.06–1.99 (m, 1H), 1.53 (s, 9H), 1.51 (s, 9H) (Supplementary Material Figure S3); ^13^C-NMR (100 MHz, CDCl_3_) δ 155.0, 154.0, 137.0, 136.8, 129.2, 129.2, 128.5, 128.5, 126.7, 126.6, 83.5, 82.5, 82.5, 81.9, 81.5, 79.8, 41.8, 41.0, 39.2, 38.8, 28.3, 28.2 (Supplementary Material Figure S4); HR-MS (C_30_H_42_N_2_O_8_Na) calcd. 581.2833 ([M + Na]^+^), Found 581.2798. Compound **3** was further characterized by heteronuclear multiple quantum coherence (HMQC) and heteronuclear multiple bond correlation (HMBC).

### 3.5. Synthesis of (3R,5S)-Tert-butyl 3-benzyl-5-hydroxyisoxazolidine-2-carboxylate *(**4a**)*

(*E*)-4-phenylbut-2-enal **1a** (146 mg, 1 mmol) was dissolved in dichloromethane (5 mL), and then *N*-Boc-hydroxylamine **2** (199 mg, 1.5 mmol), (*S*)-diphenylprolinol-TMS II (33 mg, 0.1 mmol), and *p*-nitrobenzoic acid (16 mg, 0.1 mmol, 10 mol %) were added to the resulting solution at 0 °C. The reaction mixture was stirred for 15 h at 0 °C. The mixture solution was diluted with dichloromethane (20 mL) and washed with saturated sodium bicarbonate aqueous solution (5 mL) and brine (5 mL). The resulting organic phase was dried over Na_2_SO_4_, filtered and concentrated under reduced pressure. The crude product was purified over silica gel column chromatography (PE/EtOAc, 10:1–4:1) to give 4a as a white solid (251 mg, 90%). mp: 138–139 °C; [α]^D^_22_ +16.50 (c 1.0, CHCl_3_); ^1^H-NMR (400 MHz, CDCl_3_) δ 7.29–7.18 (m, 5H), 5.71 (d, *J* = 4.4 Hz, 1H), 4.49–4.42 (m, 1H), 3.00 (dd, *J* = 8.3 Hz, 12 Hz, 1H), 2.70 (dd, *J* = 8.3 Hz, 12 Hz, 1H), 2.28 (dd, *J* = 8.4 Hz, 12 Hz, 1H), 2.00–1.94 (m, 1H), 1.35 (s, 9H) (Supplementary Material Figures S5 and S7); ^13^C-NMR (100 MHz, CDCl_3_) δ 158.8, 138.2, 129.4, 128.3, 126.4, 98.7, 82.0, 59.4, 42.1, 41.3, 28.0 (Supplementary Material Figures S6 and S8); HR-MS (C_15_H_21_NO_4_Na) calcd. 302.1363 ([M + Na]^+^), Found 302.1370; 92% ee; Chiral HPLC condition: SHIMADZU Essentia LC-16 HPLC, Chiralcel AD-H column (250 × 4.6 mm, i.d.) with a mixture of hexane and 2-propanol (95:5) at a flow rate of 1.0 mL/min as the mobile phase, oven temperature was 28 °C, 210 nm, tminor = 6.66 min, tmajor = 6.00 min. A quintuple scale of this reaction was also carried out and a similar result was observed (88% yield, 92% ee).

### 3.6. Synthesis of (3R)-Tert-butyl 3-benzyl-5-(tert-butoxycarbonyloxy)isoxazolidine-2-carboxylate *(**5**)*

(*E*)-4-phenylbut-2-enal **1a** (146 mg, 1 mmol) was dissolved in dichloromethane (5 mL), and then *N*-Boc-hydroxylamine **2** (199 mg, 1.5 mmol), (*S*)-diphenylprolinol-TMS II (33 mg, 0.1 mmol), and *p*-Toluenesulfonic acid (34 mg, 0.2 mmol, 10 mol %) were added to the resulting solution at 0 °C. The reaction mixture was stirred for 8 h at 0 °C. The mixture solution was diluted with dichloromethane (20 mL) and washed with saturated sodium bicarbonate aqueous solution (5 mL) and brine (5 mL). The resulting organic phase was dried over Na_2_SO_4_, filtered, and concentrated under reduced pressure. The crude product was purified over silica gel column chromatography (PE/EtOAc, 10:1–4:1) to give **5** as a wax solid (246 mg, 65%); [α]^D^_22_ +13.80 (c 1.0, CHCl_3_); ^1^H-NMR (400 MHz, CDCl_3_) δ 7.30–7.20 (m, 5H), 6.11–6.08 (m, 1H), 4.62–4.55 (m, 1H), 2.99 (dd, *J* = 8.6 Hz, 12 Hz, 1H), 2.71 (dd, *J* = 8.5 Hz, 12 Hz, 1H), 2.63–2.57 (m, 1H), 2.25–2.19 (m, 1H), 1.50 (s, 9H), 1.39 (s, 9H) (Supplementary Material Figure S11); ^13^C-NMR (100 MHz, CDCl_3_) δ 158.3, 155.6, 137.7, 129.3, 128.3, 126.4, 88.5, 82.8, 82.2, 61.2, 41.3, 35.2, 28.1, 27.9; HR-MS (C_20_H_29_NO_6_Na) calcd. 402.1893 ([M + Na]^+^), Found 402.1891. 43% ee; Chiral HPLC condition: SHIMADZU Essentia LC-16 HPLC, Chiralcel AD-H column (250 × 4.6 mm, i.d.) with a mixture of hexane and 2-propanol (95:5) at a flow rate of 1.0 mL/min as the mobile phase, oven temperature was 28 °C, 210 nm, tminor = 10.02 min, tmajor = 8.08 min.

### 3.7. Synthesis of (3R,5S)-Tert-butyl 5-hydroxy-3-(2,4,5-trifluorobenzyl)isoxazolidine-2-carboxylate *(**4b**)*

(*E*)-4-(2,4,5-trifluorophenyl)but-2-enal (200 mg, 1 mmol) was dissolved in dichloromethane (5 mL), and then *N*-Boc-hydroxylamine **2** (159 mg, 1.2 mmol), (*S*)-diphenylprolinol-TMS II (33 mg, 0.1 mmol), and p-nitrobenzoic acid (16 mg, 0.1 mmol, 10 mol %) were added to the resulting solution at −20 °C. The reaction mixture was stirred for 15 h at −20 °C. The mixture solution was diluted with dichloromethane (20 mL) and washed with saturated sodium bicarbonate aqueous solution (5 mL) and brine (5 mL). The resulting organic phase was dried over Na_2_SO_4_, filtered and concentrated under reduced pressure. The crude product was purified over silica gel column chromatography (PE/EtOAc, 10:1–4:1) to give **4b** as a white solid (283 mg, 85%). mp: 156–157 °C; [α]^D^_22_ +22.70 (c 1.0, CHCl_3_); ^1^H-NMR (400 MHz, CDCl_3_) δ 7.09–7.03 (m, 1H), 6.91–6.85 (m, 1H), 5.70 (d, *J* = 4.3 Hz, 1H), 4.51–4.44 (m, 1H), 2.80 (d, *J* = 8.8 Hz, 2H), 2.37 (dd, *J* = 8.7 Hz, 12 Hz, 1H), 1.98–1.92 (m, 1H), 1.35 (s, 9H) (Supplementary Material Figure S9); ^13^C-NMR (100 MHz, CDCl_3_) δ 158.7, 156.1, 148.8, 145.1, 121.4, 119.2, 105.1, 98.7, 82.3, 57.9, 41.2, 34.5, 27.9 (Supplementary Material Figure S10); HR-MS (C_15_H_18_F_3_NO_4_Na) calcd. 356.1086 ([M + Na]^+^), Found 356.1087; 96% ee; Chiral HPLC condition: SHIMADZU Essentia LC-16 HPLC, Chiralcel AD-H column (250 × 4.6 mm, i.d.) with a mixture of hexane and 2-propanol (95:5) at a flow rate of 1.0 mL/min as the mobile phase, oven temperature was 28 °C, 210 nm, tminor = 11.02 min, tmajor = 9.30 min.

### 3.8. Synthesis of (R)-N-Boc-3-benzyl-5-oxoisoxazolidine *(**6a**)* and (R)-N-Boc-3-trifluorobenzyl-5-oxoisoxazolidine *(**6b**)*

A solution of NaClO_2_ (127 mg, 1.4 mmol) in water (1.4 mL) was added dropwise to a stirred solution of (3*R*,5*S*)-*tert*-butyl-3-benzyl-5-hydroxyisoxazolidine-2-carboxylate **4a** (279 mg, 1 mmol, 92% ee) in acetonitrile (5 mL) and NaH_2_PO_4_ (24 mg, 0.2 mmol) in water (1 mL) and H_2_O_2_ (35 W% in water, 0.14 mL, 1.4 mmol) at 10 °C. The solution was diluted with dichloromethane (20 mL) and washed with saturated sodium bicarbonate aqueous solution (5 mL) and brine (5 mL). The resulting organic phase was dried over Na_2_SO_4_, filtered and concentrated under reduced pressure. The crude product was purified over silica gel column chromatography (PE/EtOAc, 5:1–3:1) to give (*R*)-*N*-Boc-3-benzyl-5-oxoisoxazolidine **6a** (227 mg, 82% yield) as a white solid. [α]^D^_22_ +40.10 (c 1.0, CHCl_3_); ^1^H-NMR (400 MHz, CDCl_3_) δ 7.22–7.30 (m, 5H), 4.58–4.65 (m, 1H), 3.04 (dd, *J* = 8.5 Hz, 12 Hz, 1H), 2.74–2.85 (m, 2H), 2.57 (dd, *J* = 4.7 Hz, 16 Hz, 1H), 1.27 (s, 9H). Spectroscopic data are in agreement with reference [22]. 92% ee; Chiral HPLC condition: SHIMADZU Essentia LC-16 HPLC, Chiralcel AD-H column (250 × 4.6 mm, i.d.) with a mixture of hexane and 2-propanol (95:5) at a flow rate of 1.2 mL/min as the mobile phase, oven temperature was 28 °C, 210 nm, tminor = 14.91 min, tmajor = 11.82 min.

Prepared from **4b** (333 mg, 1 mmol), according to the procedure of the synthesis of **6a**, **6b** was obtained as a white solid (109 mg, 33%). ^1^H-NMR (400 MHz, CDCl_3_) δ 7.29–7.18 (m, 5H), 5.71 (d, *J* = 4.1 Hz, 1H), 4.49–4.42 (m, 1H), 3.00 (dd, *J* = 8.4 Hz, 12 Hz, 1H), 2.70 (dd, *J* = 8.5 Hz, 12 Hz, 1H), 2.28 (dd, *J* = 8.4 Hz, 12 Hz, 1H), 2.00–1.94 (m, 1H), 1.35 (s, 9H); ^13^C-NMR (100 MHz, CDCl_3_) δ 173.1, 158.2, 156.7, 148.1, 146.6, 123.4, 121.9, 107.1, 82.8, 59.2, 42.3, 36.1, 28.9; HR-MS (C_15_H_16_F_3_NO_4_Na) calcd. 354.0929 ([M + Na]^+^), Found 354.0927. 96% ee; Chiral HPLC condition: SHIMADZU Essentia LC-16 HPLC, Chiralcel AD-H column (250 × 4.6 mm, i.d.) with a mixture of hexane and 2-propanol (95:5) at a flow rate of 1.2 mL/min as the mobile phase, oven temperature was 28 °C, 210 nm, tminor = 15.31 min, tmajor = 12.72 min.

### 3.9. Synthesis of (R)-N-Boc-β-benzyl-β-amino acid 7a and (R)-N-Boc-β-(2,4,5-trifluorobenzyl)-β-amino acid ***7b*** via oxidation/reductive cleavage of N-O bond sequence

To a solution of (*R*)-*N*-Boc-3-benzyl-5-oxoisoxazolidine **6a** (277 mg, 1 mmol, 92% ee) in MeOH (15 mL), Pd/C (20% *w*/*w*, 55 mg) was added. The reaction mixture was hydrogenated under 90 atm for 24 h. The reaction mixture was filtered through celite with ethyl acetate and concentrated to give (*R*)-*N*-Boc-*β*-benzyl-*β*-amino acid **7a** (276 mg, 99%) as a white solid. mp: 111–113 °C; [α]^D^_22_ +18.10 (c 1.0, CHCl_3_); ^1^H-NMR (400 MHz, CDCl_3_) δ 7.33–7.24 (m, 5H), 5.04–4.97 (m, 1H), 2.98 (dd, *J* = 8.4 Hz, 16, 1H), 2.87–2.84 (m, 2H), 2.62 (dd, *J* = 4.3 Hz, 16 Hz, 1H), 1.30 (s, 9H) (Supplementary Material Figure S12). Spectroscopic data are in agreement with reference [23].

Prepared from **6b** (331 mg, 1 mmol) according to the procedure of the synthesis of **7a**, **7b** was obtained as a white solid (330 mg, 99%). mp: 121–123 °C; [α]^D^_22_ −29.80 (c 1.0, CHCl_3_) (Supplementary Material Figure S13); ^1^H-NMR (400 MHz, CDCl_3_) δ 7.12–7.08 (m, 1H), 6.96–6.90 (m, 1H), 5.09–5.07 (m, 1H), 4.18–4.15 (m, 1H), 2.92–2.90 (m, 2H), 2.65–2.57 (m, 2H), 1.41 (s, 9H); Spectroscopic data are in agreement with reference [8].

### 3.10. Synthesis of (R)-N-Boc-β-benzyl-β-amino acid ***7a*** and (R)-N-Boc-β-(2,4,5-trifluorobenzyl)-β-amino acid ***7b*** via reductive cleavage of N-O bond/oxidation sequence

#### 3.10.1. Method A

To a solution of (3*R*,5*S*)-*tert*-butyl 3-benzyl-5-hydroxyisoxazolidine-2-carboxylate **4a** (279 mg, 1 mmol, 92% ee) in CH_3_CN/H_2_O (6 mL, 9:1, degassed under nitrogen) was added Mo(CO)_6_ (1.2 equiv., 1.2 mmol). The reaction mixture was vigorously stirred and heated to reflux for 4 h. The resulting solution was then filtered through a short column of silica gel (washed by ethyl acetate). The resulting after evaporation was dissolved in dichloromethane (2 mL) and then it was cooled down to 4 °C. The resulting solution was added isobutene (1 mL), *tert*-butanol (4 mL), H_2_O (2 mL), KH_2_PO_4_ (544 mg, 4 mmol), and NaClO_2_ (360 mg, 4 mmol), sequentially. The reaction was stirred at this temperature for 12 h. The mixture was diluted with 20 W% NaOH aqueous solution (10 mL) and dichloromethane (10 mL). The organic phase was decanted. The aqueous phase was adjusted to pH = 4–5 and then extracted with dichloromethane and the combined organic phase was washed with brine, dried over Na_2_SO_4_, and evaporated to give (*R*)-*N*-Boc-*β*-benzyl-*β*-amino acid **7a** as a white solid (231 mg, 83%).

(*R*)-*N*-Boc-*β*-(2,4,5-trifluorobenzyl)-*β*-amino acid **7b** was prepared from **4b** (333 mg, 1 mmol) according to the procedure of the synthesis of **7a**, and **7b** was obtained as a white solid (273 mg, 82%).

#### 3.10.2. Method B

To a solution of (3*R*,5*S*)-*tert*-butyl 3-benzyl-5-hydroxyisoxazolidine-2-carboxylate **4a** (279 mg, 1 mmol, 92% ee) in methanol (10 mL), NaBH_4_ (75.6 mg, 1 mmol) was added carefully at 4 °C, and the reaction mixture was allowed to stir at room temperature for 8 h, and then evaporated. The residue was diluted with water (20 mL), and then the water phase was extracted with dichloromethane. The combined organic phase was washed with brine, dried over Na_2_SO_4_, and evaporated to give a liquid. The obtained liquid was dissolved in methanol (20 mL) and the reaction mixture was hydrogenated with Raney Ni (100 mg) at normal pressure for 8 h. The resulting solution was filtered and evaporated. The residue was purified over silica gel column to give (*R*)-*tert*-butyl 4-hydroxy-1-phenylbutan-2-ylcarbamate **9a** as white solid (220 mg, 83%). Compound **9a** (220 mg, 1.5 mmol) was dissolved in the mixture solution of dichloromethane (11 mL) and aqueous sodium bicarbonate (2.5 mL, 5% *w*/*w*). 2,2,6,6-tetramethylpiperidine-1-oxyl (TEMPO) (13.2 mg, 0.08 mmol) and sodium bromide (8.5 mg, 0.08 mmol) were added to this reaction mixture and it was cooled down to 0 °C. Sodium hypochlorite solution (3.7 mL, 2.5 mmol, 5% *w*/*w*) was added dropwise, and then the reaction mixture was stirred for 3 h. The reaction mixture was quenched with saturated aqueous thiosulfate solution (1 mL), followed by adding aqueous hydrochloric acid (2 M) until the pH = 4–5. The aqueous phase was extracted with dichloromethane (10 mL × 3). The combined organic phase was dried over Na_2_SO_4_ and concentrated to give compound **7a** as a white solid (206 mg, 89%).

(*R*)-*N*-Boc-β-(2,4,5-trifluorobenzyl)-*β*-amino acid **7b** was prepared from **4b** (333 mg, 1 mmol) according to the procedure of the synthesis of **7a**, and **7b** was obtained as a white solid (256 mg, 77% over two steps).

#### 3.10.3. Method C

To a solution of (3*R*,5*S*)-*tert*-butyl 3-benzyl-5-hydroxyisoxazolidine-2-carboxylate **4a** (500 mg, 1.79 mmol, 92% ee) in MeOH (10 mL), Pd/C (20% *w*/*w*, 0.1 g) was added. The reaction mixture was hydrogenated under 90 atm for 24 h. The reaction mixture was filtered through celite with ethyl acetate and concentrated to give (*R*)-*tert*-butyl 4-hydroxy-1-phenylbutan-2-ylcarbamate **9a** as a white solid (474 mg, 99%), which (474 mg, 1.79 mmol) was dissolved in the mixture solution of dichloromethane (24 mL) and aqueous sodium bicarbonate (5.4 mL, 5% *w*/*w*). TEMPO (28.8 mg, 0.18 mmol) and sodium bromide (18.6 mg, 0.18 mmol) were added to this reaction mixture and it was cooled down to 0 °C. Sodium hypochlorite solution (8 mL, 5.4 mmol, 5% *w*/*w*) was dropwise added, and then the reaction mixture was stirred for 3 h. The reaction mixture was quenched with saturated aqueous thiosulfate solution (2 mL), followed by adding aqueous hydrochloric acid (2 M) until the pH = 4–5. The aqueous phase was extracted with dichloromethane (20 mL × 3). The combined organic phase was dried over Na_2_SO_4_ and concentrated to give compound **7a** as a white solid (440 mg, 89%).

(*R*)-*N*-Boc-*β*-(2,4,5-trifluorobenzyl)-*β*-amino acid **7b** was prepared from **4b** (333 mg, 1 mmol) according to the procedure of the synthesis of **7a,** and **7b** was obtained as a white solid (300 mg, 90%).

### 3.11. Synthesis of (R)-tert-butyl 4-oxo-1-phenylbutan-2-ylcarbamate *(**8a**)* and Synthesis of (R)-tert-butyl 4-oxo-1-2,4,5-trifluorobenzylbutan-2-ylcarbamate *(**8b**)*

To a solution of (3*R*,5*S*)-*tert*-butyl 3-benzyl-5-hydroxyisoxazolidine-2-carboxylate **4a** (112 mg, 0.4 mmol, 92% ee) in CH_3_CN/H_2_O (4.0 mL, 9:1, degassed under nitrogen), Mo(CO)_6_ (126.7 mg, 0.48 mmol, 1.2 equiv.) was added. The reaction was stirred and heated to reflux for 8 h. The reaction mixture was filtered through a short column of silica gel (washed by EtOAc). The obtained solution was evaporated and purified over silica gel column chromatography (PE/EtOAc, 5:1) to give compound 8a as a white solid. 89% yield (93 mg); [α]^D^_22_ +18.10 (c 1.0, CHCl_3_); ^1^H-NMR (400 MHz, CDCl3) δ 9.69 (s, 1H), 7.31–7.21 (m, 3H), 7.15 (d, *J* = 8.2 Hz, 2H), 4.75 (s, 1H), 4.25 (s, 1H), 2.95–2.90 (m, 1H), 2.79 (dd, *J* = 8.2 Hz, 12 Hz, 1H), 2.63–2.48 (m, 2H), 1.30 (s, 9H) [24].

(*R*)-*tert*-butyl 4-oxo-1-2,4,5-trifluorobenzylbutan-2-ylcarbamate **8b** was prepared from **4b** (333 mg, 1 mmol) according to the procedure of the synthesis of **8a**, **8b** was obtained as a white solid (276 mg, 87%): ^1^H-NMR (400 MHz, DMSO-*d*_6_) δ 9.60 (s, 1H), 7.43–7.48 (m, 1H), 7.30–7.32 (m, 1H), 6.88 (d, *J* = 4.7 Hz, 1H), 4.15–4.17 (m, 1H), 2.55–2.98 (m, 2H), 2.50–2.53 (m, 2H), 1.27 (s, 9H) [25].

### 3.12. Synthesis of (R)-Tert-butyl 4-hydroxy-1-phenylbutan-2-ylcarbamate *(**9a**)* and (R)-tert-butyl 4-hydroxy-1-2,4,5-trifluorobenzylbutan-2-ylcarbamate *(**9b**)*

To a solution of (3*R*,5*S*)-*tert*-butyl 3-benzyl-5-hydroxyisoxazolidine-2-carboxylate **4a** (500 mg, 1.79 mmol, 92% ee) in MeOH (10 mL), Pd/C (20% *w*/*w*, 0.1 g) was added. The reaction mixture was hydrogenated under 90 atm for 24 h. The reaction mixture was filtered through celite with ethyl acetate and concentrated to give compound **9a** as a white solid. 99% yield (474 mg); [α]^D^_22_ −2.50 (c 1.0, CHCl_3_); ^1^H-NMR (400 MHz, CDCl_3_) δ 7.34–7.20 (m, 5H), 4.49 (d, *J* = 12.1 Hz, 1H), 4.15–4.07 (m, 1H), 3.67 (s, 2H), 3.18–3.12 (m, 1H), 2.83 (d, *J* = 4.7 Hz, 2H), 1.91–1.83 (m, 1H), 1.43 (s, 9H); ^13^C-NMR (100MHz, CDCl_3_) δ 156.8, 138.8, 129.3, 128.2, 126.2, 81.0, 59.6, 58.2, 38.4, 33.9, 28.1 [26].

(*R*)-*tert*-butyl 4-hydroxy-1-2,4,5-trifluorobenzylbutan-2-ylcarbamate **9b** was prepared from **4b** (333 mg, 1 mmol) according to the procedure of the synthesis of **9a**, and **9b** was obtained as a white solid (316 mg, 99%): ^1^H-NMR (400 MHz, CDCl_3_) δ 7.03–7.10 (m, 1H), 6.90–6.96 (m, 1H),4.49 (d, *J* = 12.5 Hz, 1H), 4.57 (d, *J* = 12.6 Hz, 1H), 4.00–4.06 (m, 1H), 3.69 (d, *J* = 4.2 Hz, 1H), 2.75–2.82 (m, 2H), 1.84–1.92 (m, 2H), 1.42 (s, 9H) [13].

### 3.13. Synthesis of Sitagliptin Phosphate Monohydrate

To a solution of **7b** (1 g, 3.0 mmol) in DCM (10 mL), EDCI (0.69 g, 3.6 mmol), HOBT (0.49 g, 3.6 mmol), 3-(trifluoromethyl)-5,6,7,8-tetrahydro-1,2,4-triazolo[4,3-*a*]pyrazine **10** (0.58 g, 3.0 mmol), and DIPEA (0.39 g, 3.0 mmol) were added successively. The reaction mixture was stirred for 12 h at room temperature and then H_2_O (10 mL) and ethyl acetate (20 mL) were added to the reaction mixture. The organic phase was washed with saturated aqueous NaHCO_3_ solution (5 mL), aqueous HCl solution (5 mL, 1N), brine (5 mL), and dried over with anhydrous Na_2_SO_4_. The solvent was evaporated and the residue was purified over silica gel chromatography to afford **11** as a foamy solid (1.38 g, 91%). Compound **11** (1.38 g, 2.7 mmol) was dissolved in 60 mL of saturated methanolic hydrogen chloride solution and stirred for 1 h. The solution was concentrated and partitioned between ethyl acetate (40 mL) and 1 N aqueous sodium hydroxide solution (40 mL). The aqueous layer was extracted with ethyl acetate (3 × 40 mL). The combined organic phase was evaporated and immediately dissolved in isopropanol (4 mL). The solution was maintained at 40 °C for 1 h and then cooled down to 20–15 °C. Heptane (12 mL) was added dropwise over 30 min and filtered. The wet cake was washed with 20% isopropanol in heptanes and dried over vacuo to give free base of 11 (0.89 g, 81%, >99.9% ee). The obtained solid (0.89 g, 2.2 mmol) was dissolved in isopropanol (3.8 mL) and H_2_O (1 mL) and 45% H_3_PO_4_ (2.5 mmol) were added dropwise. The mixture was heated to 70–80 °C to dissolve the solids and then cooled to 60–65 °C and seeded with trace sitagliptin phosphate monohydrate. The batch was aged for 1 h and cooled to ambient temperature. 2-Propanol (2.8 mL) was added to the batch slowly and then filtered. The wet cake was washed with aqueous isopropanol (20% water, 3 mL), and dried over vacuo at 60 °C to give sitagliptin phosphate monohydrate **1** (1.1 g, 96% yield, >99.9% ee). Chiral HPLC condition: SHIMADZU Essentia LC-16 HPLC, Chiralpak IC-3 column with a mixture of 40% hexanes (0.1% diethylamine), 60% 2-PrOH (0.1% diethylamine) at a flow rate of 0.5 mL/min as the mobile phase, oven temperature was 28 °C, 210 nm, tminor = 21.12 min, tmajor = 19.10 min. Data of sample was consistent with that of standard sample [6].

## 4. Conclusions

In summary, we disclosed an efficient and practical route for the asymmetric synthesis of sitagliptin phosphate monohydrate using chiral hemiactal as the key intermediate starting from (*E*)-4-(2,4,5-trifluorophenyl)but-2-enal. The chiral hemiacetal fragment was constructed by tandem aza-Michael/hemiacetal reaction catalyzed with organocatalyst, without the use of expensive metal catalyst. Furthermore, the influence of acidity of Brønsted acid on tandem aza-Michael/hemiacetal reaction was addressed in detail.

## Figures and Tables

**Table 1:**
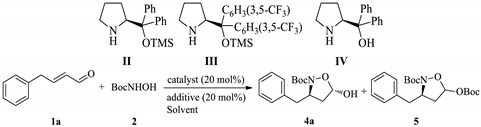
Tandem aza-Michael/hemiacetal reaction between (*E*)-4-phenylbut-2-enal **1a** and *N*-Boc-hydroxylamine at the presence of secondary amine and Brønsted acid additives ^a^.

Entry	Cat.	Solvent	Additive	Product (% Yield ^b^, % ee ^c^)
1	II	CHCl_3_	H_3_PO_4_	**4a** (21, 43) + **5** (35, 43)
2	II	CHCl_3_	TFA	**4a** (18, 41) + **5** (42, 42)
3	II	CHCl_3_	*p*-TsOH	**4a** (8, 44) + **5** (65, 43)
4	II	CHCl_3_	*p*-NO_2_C_6_H_4_COOH	**4a** (84, 92)
5	II	Et_2_O	*p*-NO_2_C_6_H_4_COOH	**4a** (78, 86)
6	II	MeOH	*p*-NO_2_C_6_H_4_COOH	**4a** (72, 76)
7	II	Toluene	*p*-NO_2_C_6_H_4_COOH	**4a** (77, 80)
8	II	DCM	*p*-NO_2_C_6_H_4_COOH	**4a** (90, 92)
9	II	DCM	*p*-NO_2_C_6_H_4_COOH	**4a** (90, 92) ^d^
10	III	DCM	*p*-NO_2_C_6_H_4_COOH	**4a** (79, 45)
11	IV	DCM	*p*-NO_2_C_6_H_4_COOH	**4a** (30, 42)

^a^ All reactions were carried out using (*E*)-4-phenylbut-2-enal **1a** (146 mg, 1 mmol), *N*-Boc-hydroxylamine **2** (199 mg, 1.5 mmol) and catalyst (0.2 mmol, 20 mol %) in solvent (5 mL) in the presence of the indicated Brønsted acid additives (20 mol %) at 0 °C for 8 h. ^b^ Isolated yield after silica-gel column chromatography. ^c^ Determined by a chiral HPLC analysis of the isolated products. ^d^ 10 mol % of (*S*)-diphenylprolinol-TMS II and 10 mol % of p-nitrobenzoic acid were applied to the reaction and the reaction was run at 0 °C for 15 h.

## Supplementary Materials

Supplementary Materials are available online.
