# Supplementary material for: Practical Asymmetric Synthesis of Sitagliptin Phosphate Monohydrate
Source: Molecules. 2018 Jun 13;23(6):1440. doi: 10.3390/molecules23061440 (PMC6099835; doi:10.3390/molecules23061440)
Supplement: Supplementary file 1 [file molecules-23-01440-s001.pdf]

Supporting Information
For
Practical Asymmetric Synthesis of Sitagliptin Phosphate Monohydrate

Hao-ling Gao[1,2], Jian-gang Yu[1], Cheng-sheng Ge[*1,2], Qun Jiang[2]

[1] College of Chemistry and Materials Engineering, Quzhou University, Quzhou 324000, China
[2] College of Chemical Engineering, Zhejiang University of Technology, Hangzhou 310014, China
* Correspondence: gechengsheng@qzu.zj.cn

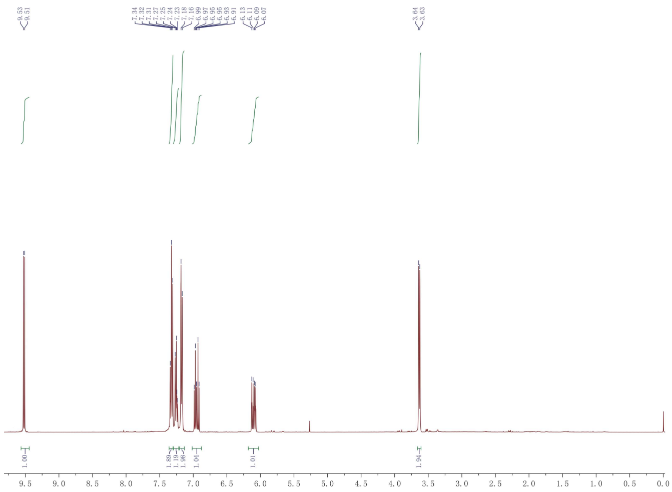

Figure S1 ^{1}H NMR spectrum (400 MHz, 297 K, $CDCl_3$) of compound **1a**

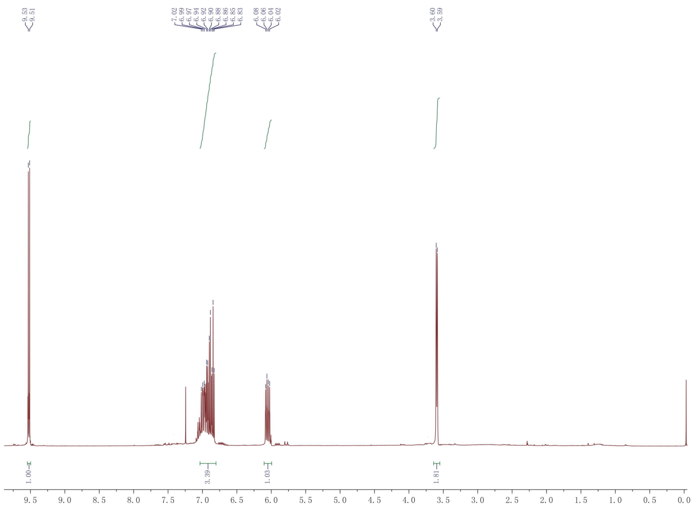

Figure S2 ^{1}H NMR spectrum (400 MHz, 297 K, $CDCl_3$) of compound **1b**

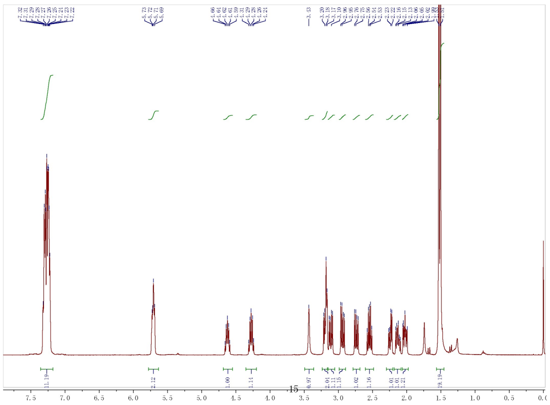

Figure S3 ^{1}H NMR spectrum (400 MHz, 297 K, $CDCl_3$) of compound **3**

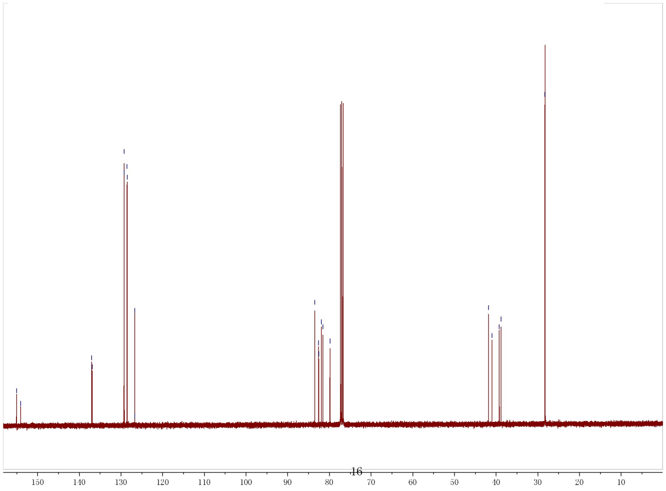

Figure S4 $^{13}C\{^{1}H\}$ NMR spectrum (100 MHz, 297 K, $CDCl_3$) of compound **3**

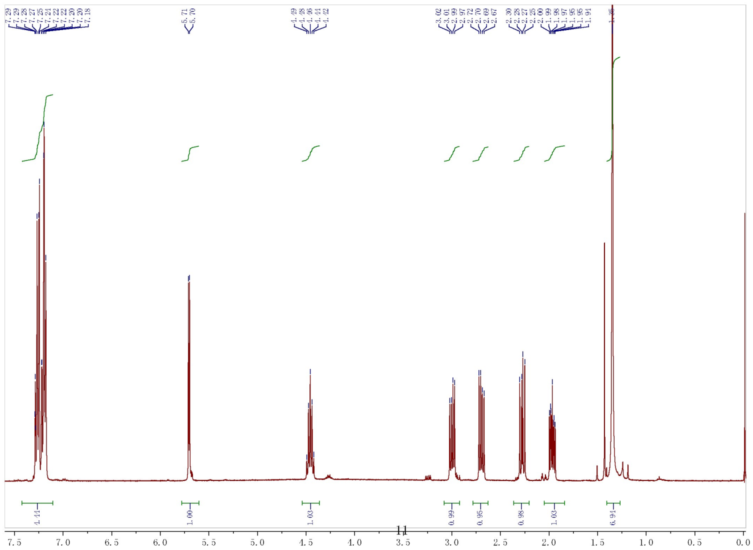

Figure S5 ^{1}H NMR spectrum (400 MHz, 297 K, $CDCl_3$) of compound **4a**

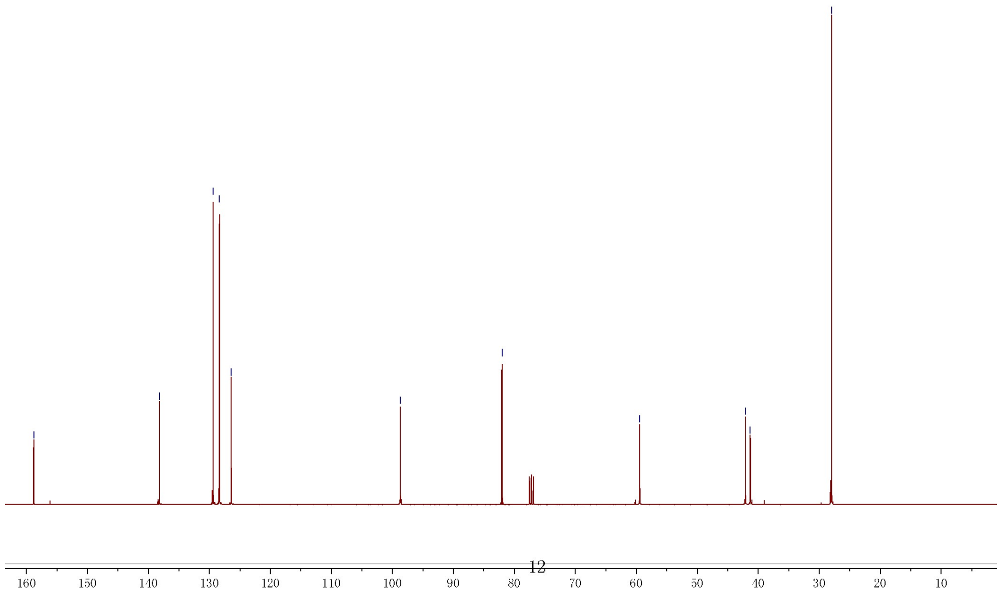

Figure S6 $^{13}C\{^{1}H\}$ NMR spectrum (100 MHz, 297 K, $CDCl_3$) of compound **4a**

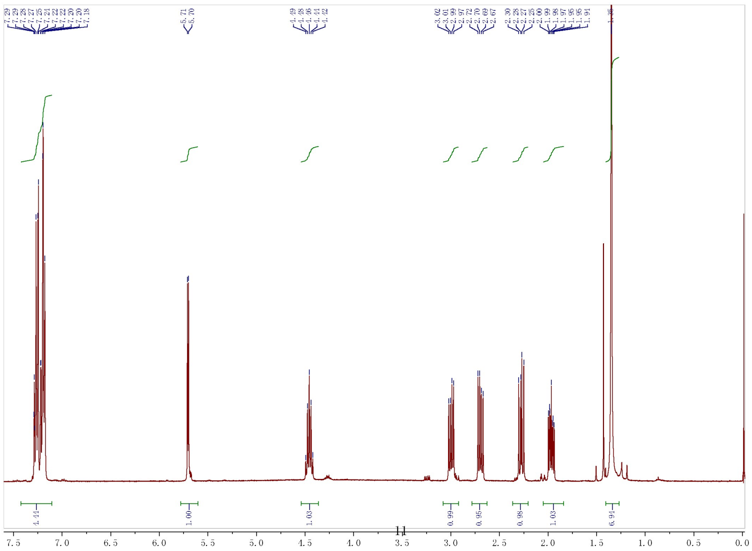

Figure S7 ^{1}H NMR spectrum (400 MHz, 297 K, $CDCl_3$) of compound **4a**

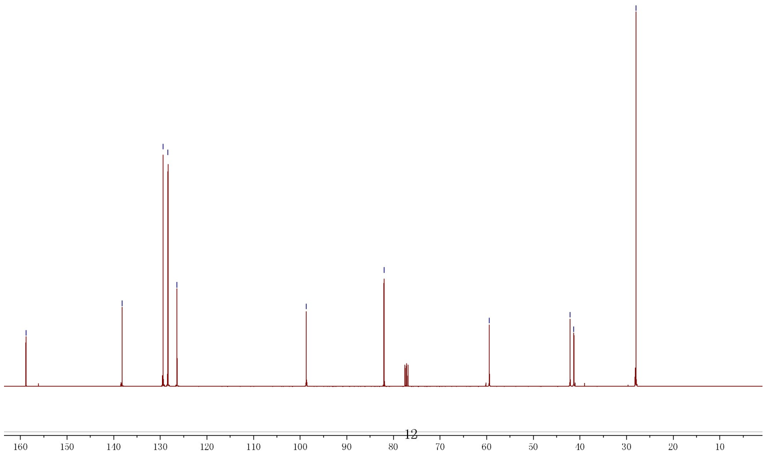

Figure S8 $^{13}C\{^{1}H\}$ NMR spectrum (100 MHz, 297 K, $CDCl_3$) of compound **4a**

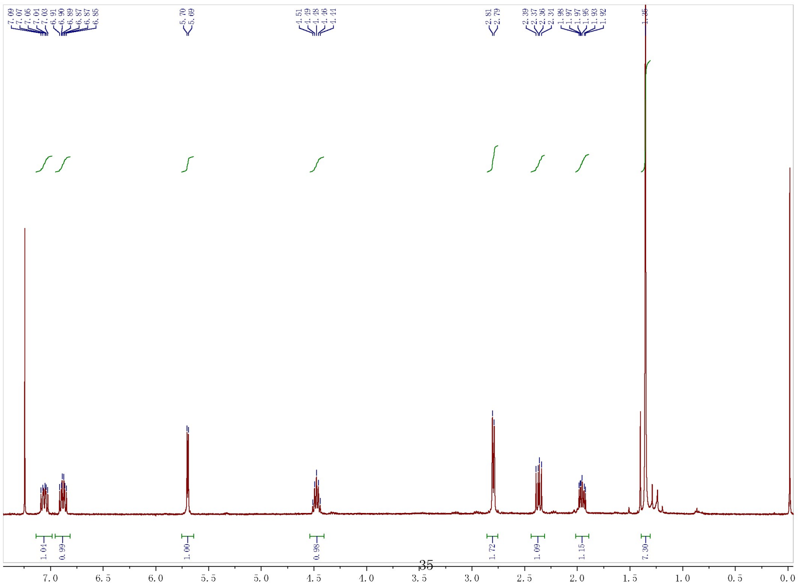

Figure S9 ^{1}H NMR spectrum (400 MHz, 297 K, $CDCl_3$) of compound **4b**

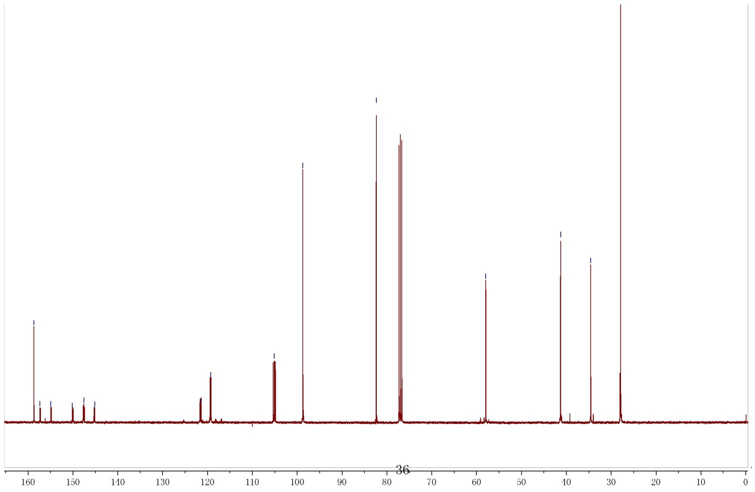

Figure S10 $^{13}C\{^{1}H\}$ NMR spectrum (100 MHz, 297 K, $CDCl_3$) of compound **4b**

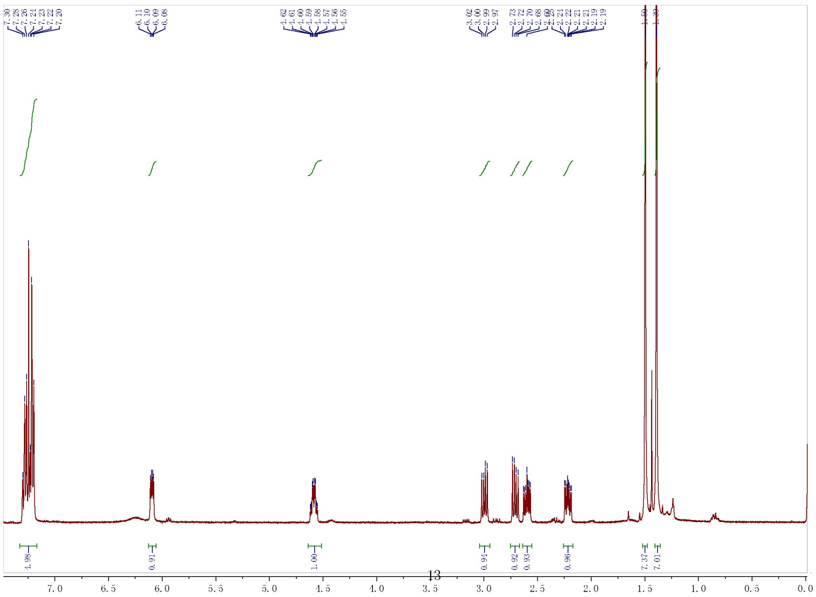

Figure S11 ^{1}H NMR spectrum (400 MHz, 297 K, $CDCl_3$) of compound **5**

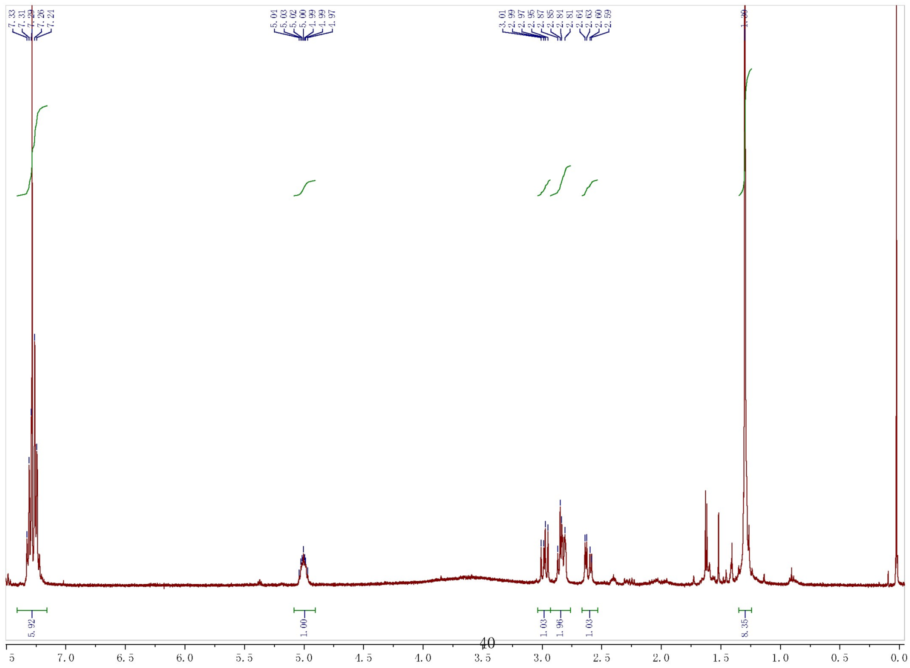

Figure S12 ^{1}H NMR spectrum (400 MHz, 297 K, $CDCl_3$) of compound **7a**

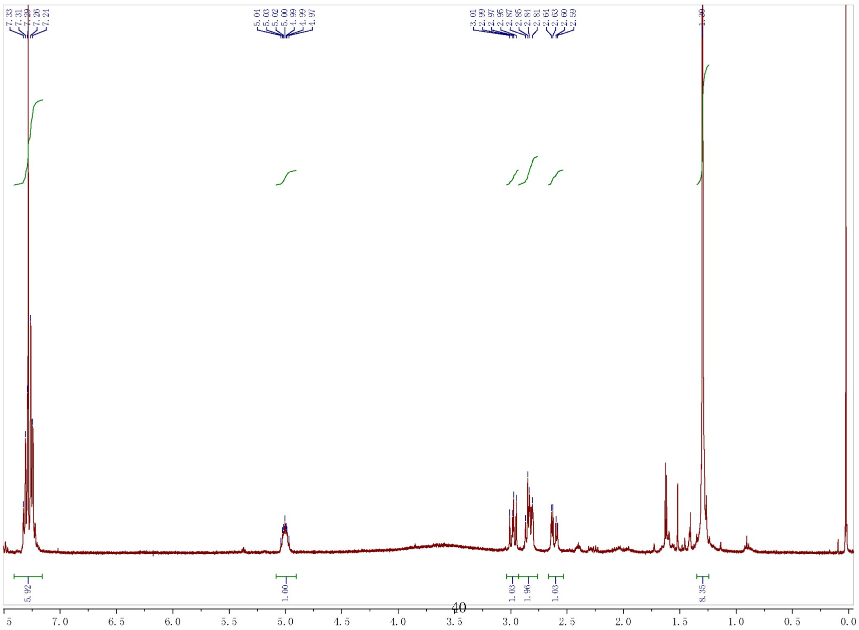

Figure S13 ^{1}H NMR spectrum (400 MHz, 297 K, $CDCl_3$) of compound **7b**

HPLC analysis

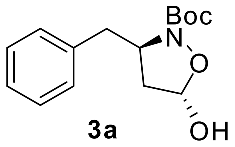 **3a**	HPLC condition: Chiralcel OD-H column, 1.0 mL/min, 5% i-PrOH/Hexane, 210 nm, t_{minor} = 6.66 min, t_{major} = 6.00 min.

Racemic **3a**

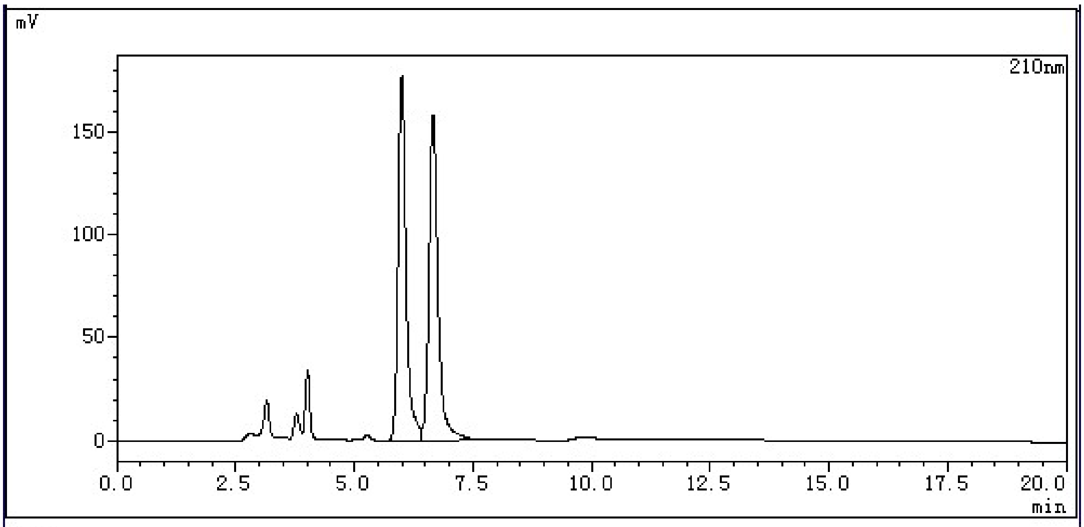

210nm

Entry	time	area	height	area %
1	6.002	2084546	177106	49.888
2	6.664	2093875	158375	50.112
		4178421	335481	100.000

(3*R*,5*S*)-tert-butyl 3-benzyl-5-hydroxyisoxazolidine-2-carboxylate (**3a**) with 92% ee

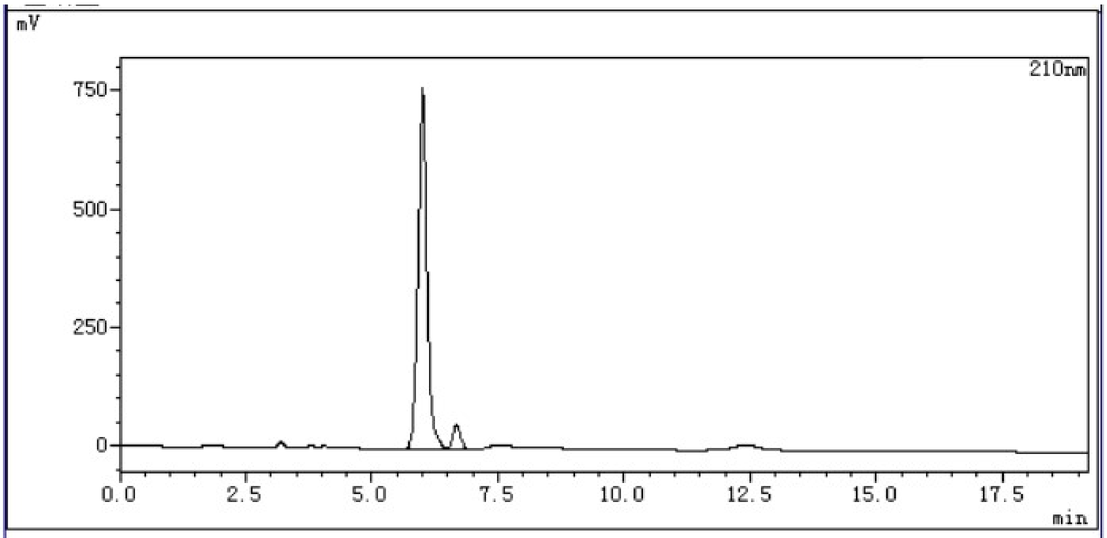

210nm

entry	time	area	height	area%
1	6.008	7883479	753350	95.988
2	6.678	329505	40198	4.012
		8212984	793548	100.000

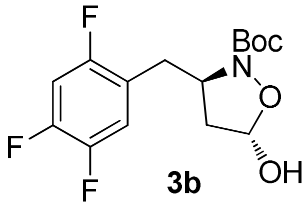<br>**3b**	HPLC condition: Chiralcel AD-H column, 1.0 mL/min, 5% i-PrOH/Hexane, 210 nm, t_{minor} = 11.02 min, t_{major} = 9.30 min.

Racemic 3j

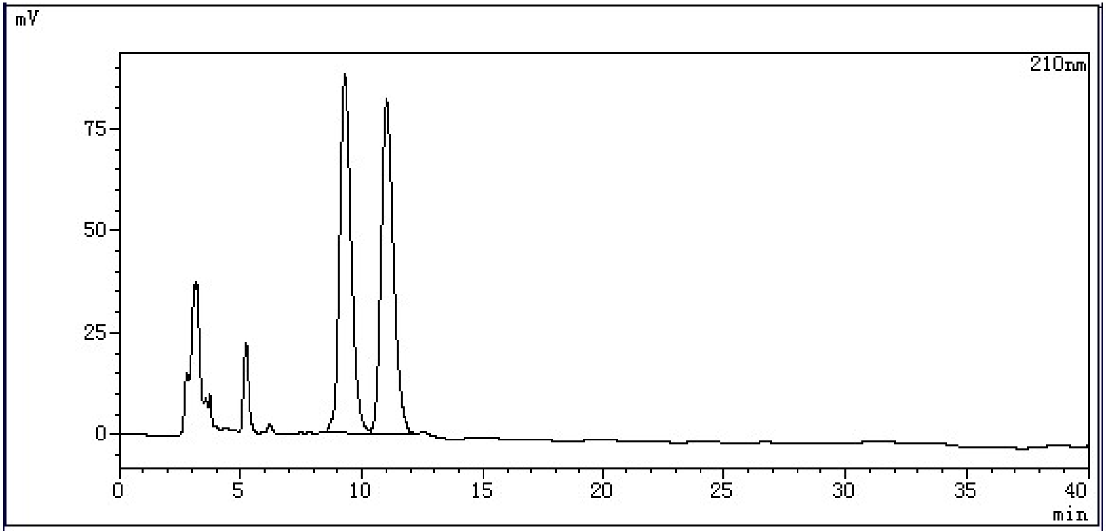

210nm

entry	time	area	height	area%
1	9.309	2951350	88011	49.896
2	11.028	2963696	82075	50.104
		5915046	170086	100.000

(3*R*,5*S*)-tert-butyl 5-hydroxy-3-(2,4,5-trifluorobenzyl)isoxazolidine-2-carboxylate (**3j**) with 96% ee

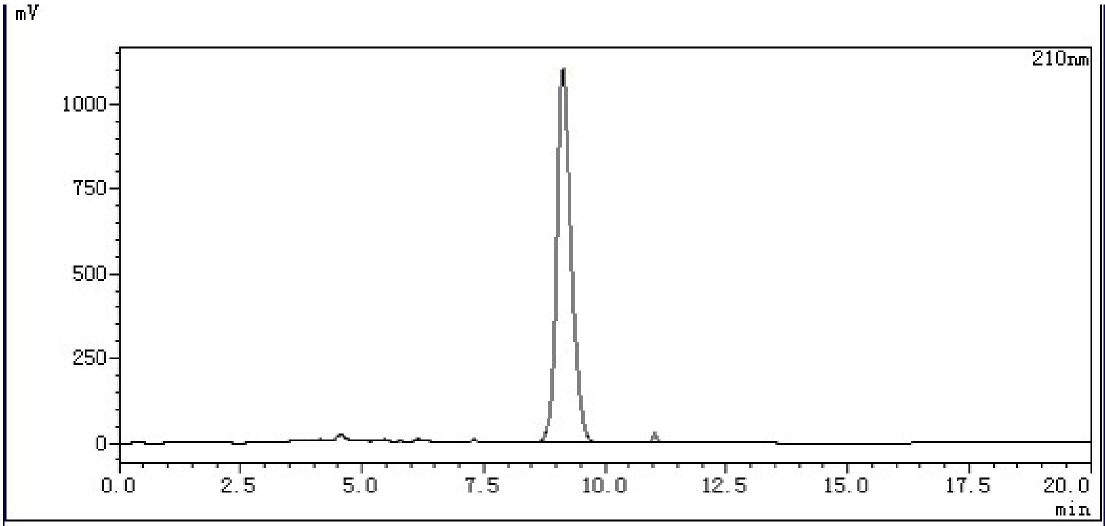

210nm

entry	time	area	height	concentration	area%
1	9.257	22779739	1102048	97.434	98.434
2	11.049	362405	32544	2.566	1.566

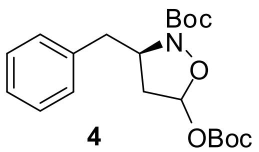 **4**	HPLC condition: Chiralcel AD-H column, 1.0 mL/min, 5% i-PrOH/Hexane, 210 nm, t_{minor} = 10.02 min, t_{major} = 8.08 min.

Racemic **4**

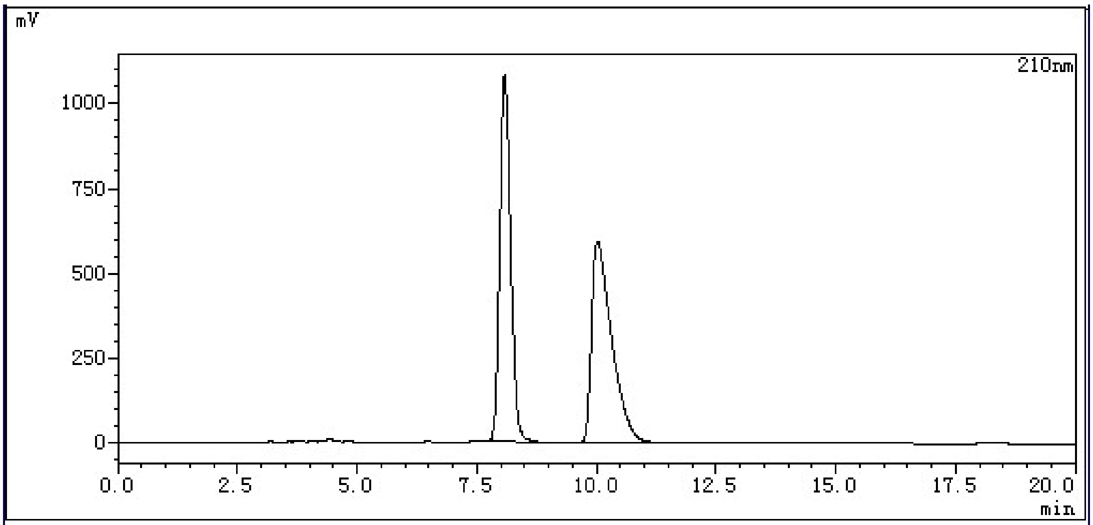

210nm

entry	time	area	height	area%
1	8.086	17126802	1077032	49.599
2	10.026	17403916	594398	50.401
		34530717	1671430	100.000

(*3R*)-tert-butyl 3-benzyl-5-(tert-butoxycarbonyloxy)isoxazolidine-2-carboxylate (**4**) with 43% ee

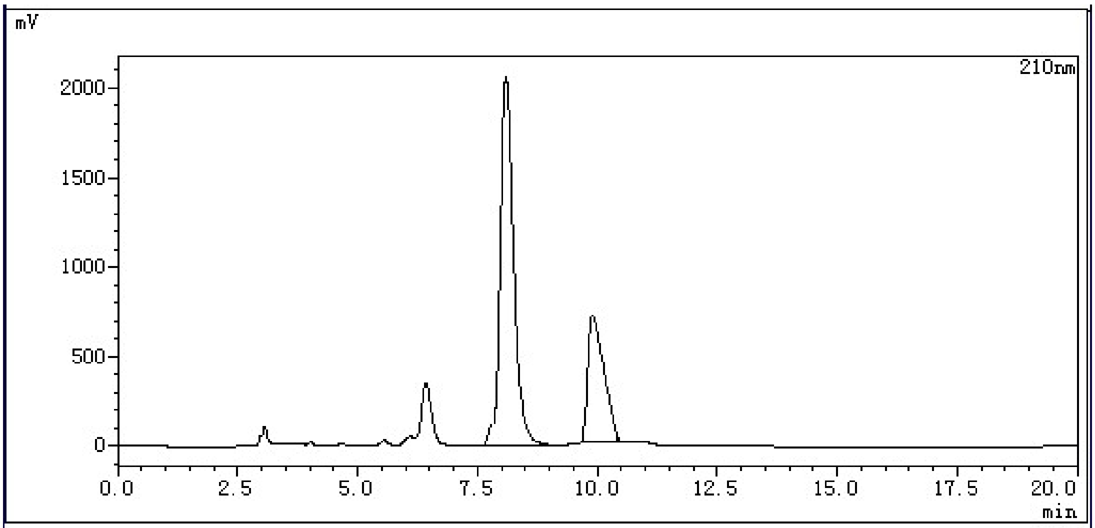

210nm

entry	time	area	height	area%
1	8.095	40860701	2047331	71.924
2	9.940	15950220	711890	28.076
		56810921	2759221	100.000

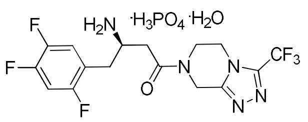

Sitagliptin phosphate monohydrate

HPLC condition: Chiralpak IC-3 column, 0.5 mL/min, 5% i-PrOH/Hexane, 210 nm, t_{minor} = 21.12 min, t_{major} = 19.10 min.

Racemic sitagliptin phosphate monohydrate

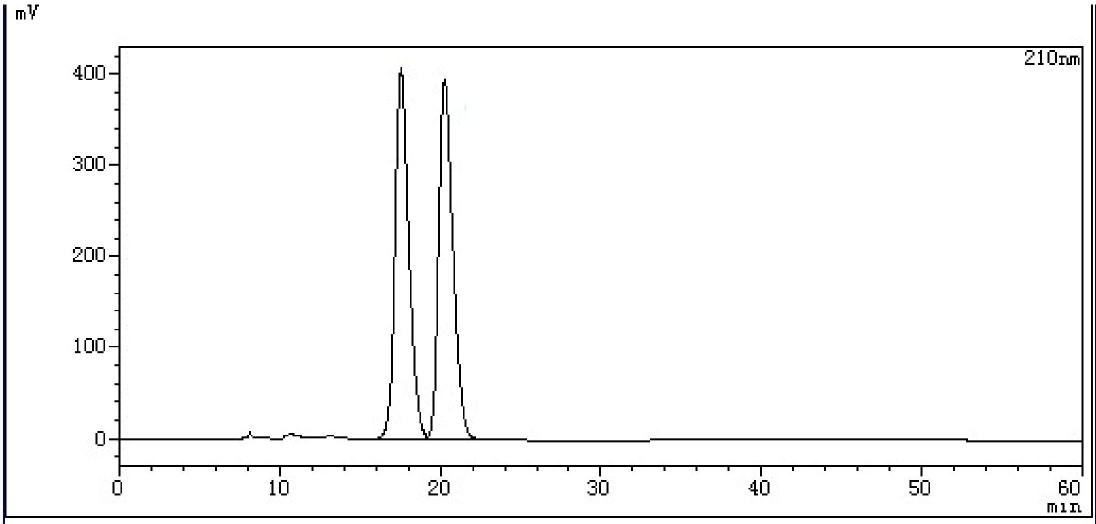

210nm

entry	time	area	height	concentration	area%
1	19.109	8067455	406275	49.974	49.974
2	21.126	8075849	394571	50.035	50.035

Sitagliptin phosphate monohydrate with >99.9% ee

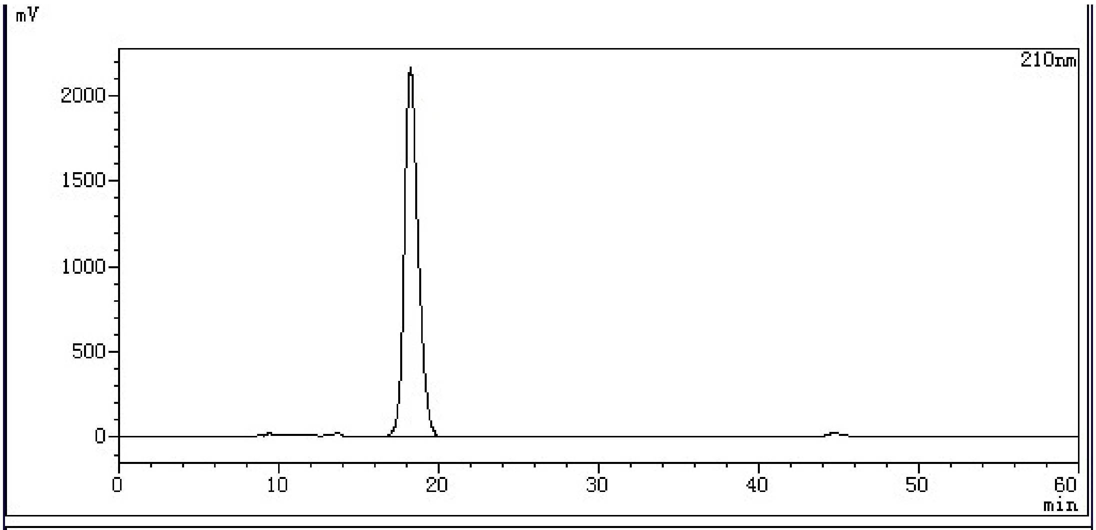

210nm

entry	time	area	height	concentration	area%
1	19.114	39661070	2141580	100.000	100.000
